# Ion mobility action spectroscopy of flavin dianions reveals deprotomer-dependent photochemistry†
†Electronic supplementary information (ESI) available: Further details of electronic structure calculations; ATDs of FAD dianions in N_2_ + ≈1% isopropyl alcohol buffer gas; photo-action ATDs for flavin mononucleotide and riboflavin monoanions; further details of photodissociation measurements. See DOI: 10.1039/c8cp03244k


**DOI:** 10.1039/c8cp03244k

**Published:** 2018-07-09

**Authors:** James N. Bull, Eduardo Carrascosa, Linda Giacomozzi, Evan J. Bieske, Mark H. Stockett

**Affiliations:** a School of Chemistry , University of Melbourne , Melbourne , VIC 3010 , Australia; b Department of Physics , Stockholm University , Stockholm , Sweden . Email: mark.stockett@fysik.su.se

## Abstract

The intrinsic optical properties and photochemistry of flavin adenine dinucleotide (FAD) dianions are investigated using a combination of tandem ion mobility spectrometry and action spectroscopy. Two principal isomers are observed, the more stable form being deprotonated on the isoalloxazine group and a phosphate (N-3,PO_4_ deprotomer), and the other on the two phosphates (PO_4_,PO_4_ deprotomer). Ion mobility data and electronic action spectra suggest that photo-induced proton transfer occurs from the isoalloxazine group to a phosphate group, converting the PO_4_,PO_4_ deprotomer to the N-3,PO_4_ deprotomer. Comparisons of the isomer selective action spectra of FAD dianions and flavin monoanions with solution spectra and gas-phase photodissociation action spectra suggests that solvation shifts the electronic absorption of the deprotonated isoalloxazine group to higher energy. This is interpreted as evidence for significant charge transfer in the lowest optical transition of deprotonated isoalloxazine. Overall, this work demonstrates that the site of deprotonation of flavin anions strongly affects their electronic absorptions and photochemistry.

## Abstract 2

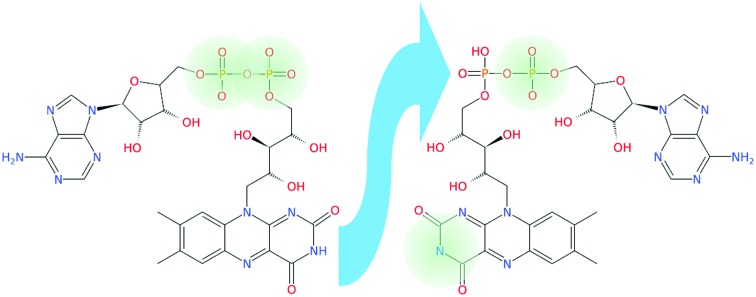
Photo-induced proton transfer, deprotomer-dependent photochemistry, and intramolecular charge transfer in flavin anions are investigated using action spectroscopy.

## 1 Introduction

Flavins are ubiquitous redox agents based on a tricyclic isoalloxazine moiety.^1^ Riboflavin (RB), flavin mononucleotide (FMN) and flavin adenine dinucleotide (FAD), which differ in the substituent at the N-10 position of isoalloxazine, occur naturally in foods including meat, cheese and beer.^2^ Flavo-enzymes and flavo-proteins containing FMN and FAD play key roles in biological functions such as DNA repair, beta-oxidation of fatty acids, and the citric acid cycle.^3^,^4^ These mechanisms take advantage of the high reduction potential of the isoalloxazine nucleus, the versatility of its several redox states, and the ability to tune the redox properties through local perturbations such as protein binding.^5^,^6^

Another important function of flavins in biochemistry is as blue light sensors regulating processes such as photosynthesis.^4^,^7^–^9^ In LOV (light, oxygen, or voltage) and BLUF (blue light sensing using FAD) domains, flavin photoreceptors control processes such as phototropism in plants^4^,^7^,^10^ and signal transduction in bacteria.^4^,^11^ These processes make use of the high photochemical activity of isoalloxazine. For example, the photocycle of FAD-containing BLUF proteins involves an excited state proton transfer between a tyrosine/protein and FAD.^4^,^12^–^15^

Although the micro-environmental sensitivity of the redox potentials of flavins is well-known,^5^,^6^ the susceptibility of their optical spectra to local perturbations has received less attention.^16^ Previous reports have found examples where flavins, especially in their deprotonated forms, have radically different absorption/emission spectra in different micro-environments.^17^–^20^ Fluorescence and molecular dynamics studies suggest FAD can exist in ‘open’ (non-π-stacked) and ‘closed’ (π-stacked) conformations in polar solvents, whereas non-polar solvents and high pH solutions (pH > 10) favour open conformations.^21^–^25^ Fluorescence experiments suggest S_1_ ← S_0_ excitation converts the closed form to the open form with a high quantum yield in the pH = 4–9 range.^21^ An understanding of the intrinsic photochemistry of flavins may be developed in a bottom-up approach starting from benchmark measurements of the transition energies and photochemical dynamics of bare molecules isolated *in vacuo*.^26^ From this starting point, incrementally more complex model systems can be investigated to quantify the impact of individual perturbations including the presence of one or several solvent molecules,^27^ charged ligands,^28^ and host–guest interactions.^29^ Gas-phase experiments are also readily compared to high-level quantum chemical calculations, which are more straightforward to carry out on isolated systems.^30^,^31^

One complication associated with flavins is the number of possible (de)protonation sites; it is not clear if the predominant site of (de)protonation in the gas phase is the same as in solution or in proteins, and what effect this may have on the optical properties.^32^,^33^ Furthermore, flavins exhibit excited state intramolecular and solvent-assisted proton transfer (phototautomerism), possibly facilitated through formation of a triplet state.^20^,^34^–^38^ There have been several earlier experimental studies on flavin ions in the gas phase,^39^–^44^ all of which have emphasised the importance of proton transfer and the challenge of assigning the most likely sites of (de)protonation. Recent photodissociation action spectroscopy measurements on FAD monoanions found indirect evidence for intramolecular proton transfer leading to the formation of the lumichrome derivative.^43^ Single wavelength photodissociation experiments have also been performed on FMN ions selected by a quadrupole mass filter^39^ or traveling wave ion mobility mass spectrometer.^45^ We are aware of only one other ion mobility study of FAD,^25^ which considered the gas-phase structures of FAD monocations, but which did not address photochemical behaviour.

**Fig. 1:**
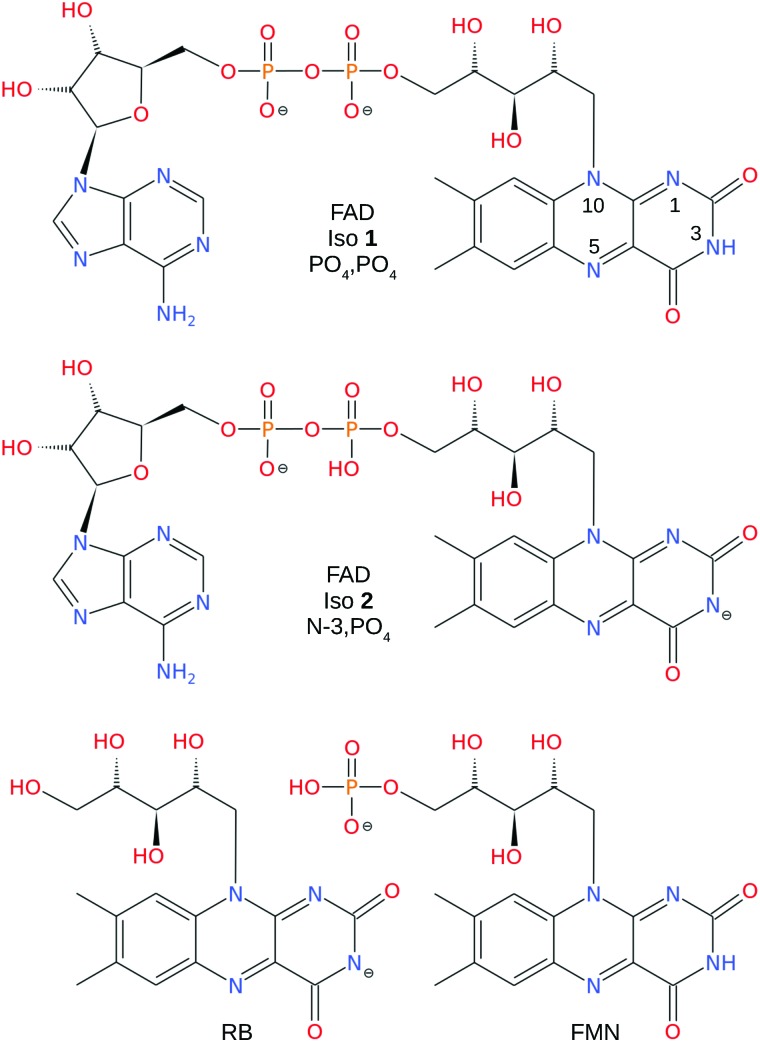
Structures of PO_4_,PO_4_ and N-3,PO_4_ deprotonated FAD dianion, N-3 deprotonated RB monoanion, and PO_4_ deprotonated FMN monoanion. The numbering scheme of the N atoms is shown on the FAD PO_4_,PO_4_ deprotomer.

In the present work, we have investigated the photochemistry of FAD dianions (Fig. 1) using tandem ion mobility spectrometry (IMS), exposing mobility-selected isomers to tunable laser light with mobility analysis of the product ions.^33^,^46^–^55^ Our results show evidence for two principal isomers, which are assigned as deprotomers based on electronic structure calculations of relative energies and collision cross-sections, and also through comparison of the action spectra with those for three different flavin monoanions, FAD, FMN and RB (see Fig. 1). Additional photodissociation measurements involving complexes of deprotonated RB and the betaine zwitterion,^56^ confirm the bright transition of deprotonated RB has strong charge-transfer character.

## 2 Methods

### 2.1 Ion mobility mass spectrometery

**Fig. 2:**
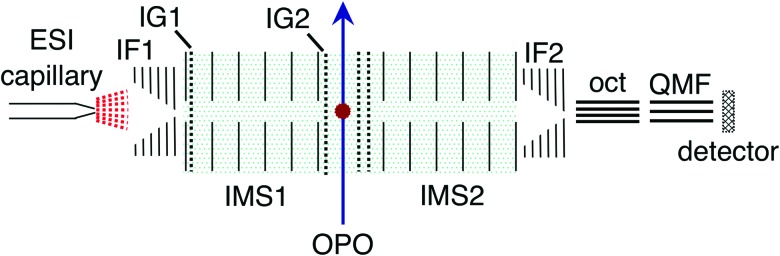
Illustration of the IMS-IMS-QMF instrument. Key: IF1 and IF2, ion funnels; IG1 and IG2, ion gates; IMS1 and IMS2, ion mobility drift regions; OPO, light beam passing through the photoisomerisation zone; oct, octupole ion guide; QMF, quadrupole mass filter. The total drift region length (IMS1 + IMS2) is 0.9 m. Figure adapted from ref. 51.

RB, FMN sodium salt, and FAD disodium salt were purchased from Sigma Aldrich (>99% purity). The experimental apparatus (illustrated in Fig. 2) has been described previously^46^–^49^ and consists of two consecutive ion mobility spectrometry drift regions followed by a quadrupole mass filter (IMS-IMS-QMF). Briefly, ions were formed by electrospray ionisation and transferred through a heated capillary into an electrodynamic ion funnel IF1 that collected the ions. The amplitude of the radio frequency (RF) potential applied to IF1 could be adjusted to heat the ions and alter the initial isomer distribution. The final electrode of the funnel (IG1) was pulsed to inject packets of ions into the first IMS stage (IMS1). The ions then drifted through N_2_ buffer gas at ∼7 Torr pressure under the influence of a 44 V cm^–1^ electric field. A second, Bradbury–Nielsen ion gate (IG2) was pulsed open to allow ions with a narrow range of collision cross-sections to enter the second IMS stage (IMS2). Immediately following the second gate, the mobility-selected ion packet was overlapped with light from an OPO laser system (EKSPLA NT342B, 20 Hz) in a crossed beam geometry. Measurements were performed with an unfocused beam and pulse energy of less than 2 mJ cm^–2^, chosen to avoid multiphoton contributions. For RB monoanions and FAD dianions under these conditions less than ∼10% of the population was depleted. The laser was operated at 20 Hz while ion packets were injected at twice this rate, allowing accumulation of ‘laser-on’ and ‘laser-off’ signals. Following photoexcitation, daughter ions were separated according to their mobility in IMS2, collected with a second ion funnel IF2 and transmitted through an octupole ion guide (oct) and a quadrupole mass filter (QMF) tuned to the parent *m*/*z*. Finally, ions were detected using a channeltron connected to a multichannel scalar, which generated a histogram of counts against arrival time, giving an arrival time distribution (ATD). The action spectra were obtained by taking the difference between ‘laser-on’ and ‘laser-off’ ATDs against laser wavelength (termed photo-action ATDs), normalised with respect to laser pulse energy and total laser-off signal. It is important to bear in mind that the photo-excitation occurs in an environment of relatively high pressure leading to collisional quenching within a few tens of nanoseconds. Slower ground-state statistical processes such as photodissociation may be suppressed.^54^

### 2.2 Photodissociation action spectroscopy

Photodissociation experiments on deprotonated RB monoanions and betaine complexes were performed using the SepI accelerator mass spectrometer at Aarhus University.^57^,^58^ Briefly, ions were electrosprayed and stored in an octupole ion trap that was emptied every 25 ms (40 Hz repetition rate). Ion bunches extracted from the octupole trap were accelerated to kinetic energies of 50 keV and the ions of interest were selected using a bending magnet. A nanosecond-pulsed laser system (EKSPLA NT342A, 20 Hz) was used to excite every second ion bunch. Measurements were performed with an unfocused beam and typical pulse intensity of <50 mJ cm^–2^. Daughter ions were separated using an electrostatic energy analyser situated after the laser-ion interaction region and counted with a channeltron detector. The difference in counts between the ‘laser-on’ and ‘laser-off’ injections provided the photo-induced signal. Unlike the IMS experiments, photo-excitation takes place in the absence of a buffer gas, enabling the observation of multi-photon induced dissociation events occurring up to 10 μs after excitation. Additional experimental details are given in the ESI.†

### 2.3 Computational

Electronic structure calculations were performed using the Gaussian 16 and MRCC (April 2017 release) software packages.^59^,^60^ Candidate deprotomer geometries where sampled using a Monte Carlo algorithm, followed by geometry optimisation using a PM6 Hamiltonian.^61^ A selection of the lowest energy geometries were reoptimised at the ωB97X-D/6-31+G(d) level of theory.^62^–^64^ Analysis of vibrational frequencies ensured these geometries were potential energy minima and provided zero point energy corrections. For FAD dianions, the geometry sampling was not meant to be exhaustive, rather suggestive of the predominant gas-phase conformation for each deprotomer. Excitation wavelengths for all species were computed at the df-CC2/6-31+G(d) level of theory and oscillator strengths were taken from CIS wavefunctions.^65^ Note that at 298 K the average internal vibrational energy of FAD dianions estimated using a harmonic oscillator partition function is 115 kJ mol^–1^ (70 kJ mol^–1^ for deprotonated RB monoanions), exceeding the energy differences between most conformations (rotations about single bonds) along the ribityl chain and the expected interconversion barriers between these conformations.

Collision cross-sections were calculated using MOBCAL with the trajectory method parameterised for N_2_ buffer gas.^66^,^67^ Input charge distributions were computed with the Merz-Singh-Kollman scheme constrained to reproduce the electric dipole moment at the ωB97X-D/6-31+G(d) level of theory.^68^ Sufficient trajectories were computed to give standard deviations of ±1 Å^2^ for the calculated values. Note that the present version of MOBCAL was parameterised for cations and its performance for monoanions or dianions has not been benchmarked.

## 3 Results and discussion

### 3.1 ATDs and deprotomer assignments

**Fig. 3:**
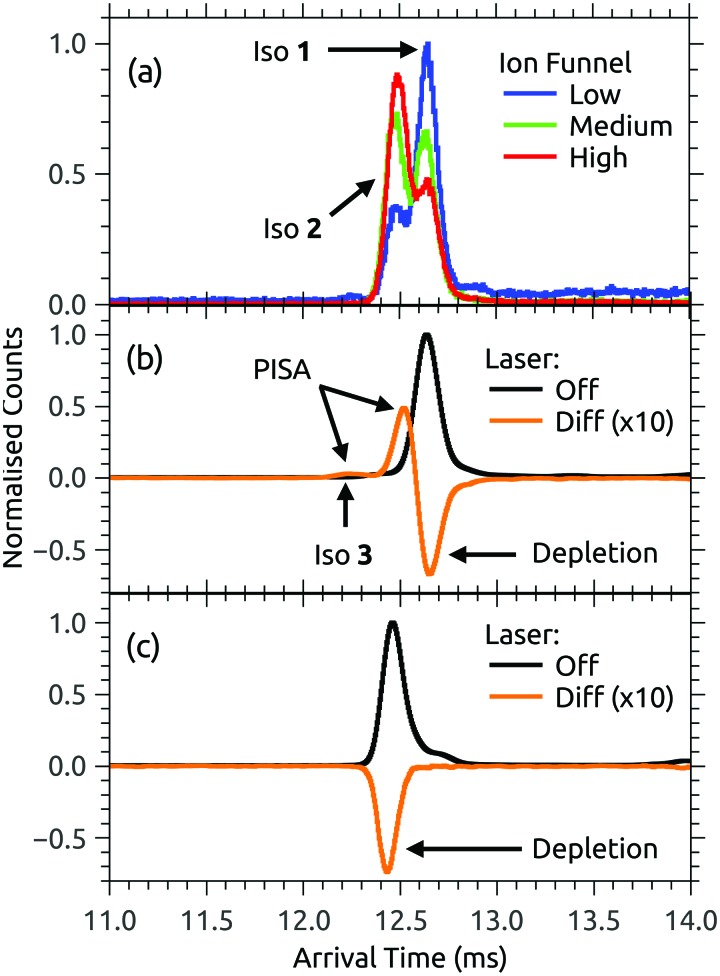
(a) ATDs for FAD dianions under different conditions – ‘low’, ‘medium’ and ‘high’ correspond to the RF drive voltage applied to IF1. A high drive voltage causes thermal isomerisation of the ions before injection into the drift region. (b) ATD for isomer **1** ions selected by IG2 (black trace) and laser on/off difference ATD for isomer **1** ions taken at 470 nm (orange). (c) ATD for isomer **2** ions selected by IG2 (black trace) and laser on/off difference or photo-action ATD for isomer **2** ions taken at 490 nm (orange). In (c), the laser was timed to interact with the short arrival time edge of the isomer **2** ion packet to minimise any contribution from isomer **1**. Net signal depletion in the difference (photo-action) ATDs is due to electron detachment. Isomer **1** is assigned to the PO_4_,PO_4_ deprotomer and isomer **2** to the N-3,PO_4_ deprotomer – see further discussion in the text.

ATDs for FAD dianions (*m*/*z* = 391.8) are shown in Fig. 3. Panel (a) shows ATDs recorded with different conditions in the first ion funnel (IF1), without using IG2 (all isomers produced by the ion source are transmitted). Two main peaks, labelled isomers **1** and **2**, are observed with similar arrival times. This is consistent with electronic structure calculations which find two low energy FAD dianion deprotomers (see Fig. 1): one with deprotonation on both phosphate groups, denoted PO_4_,PO_4_, and a second isomer with deprotonation on the isoalloxazine N-3 and a phosphate group, denoted N-3,PO_4_, which is slightly (2 kJ mol^–1^) lower in energy. With low RF drive voltage applied to the source ion funnel (IF1), minimal heating of the ions is induced by collisions with the background gas. As the RF drive voltage is increased, the ions are heated, promoting collision-induced isomerisation toward the more stable gas-phase structure,^33^,^49^,^51^ implying that isomer **2**, which increases in relative intensity with RF voltage, is lower in energy.

Additional ATDs were obtained whereby a small amount of isopropyl alcohol was added to the buffer gas to help resolve the two deprotomers (see ESI†).^54^,^55^ These ATDs show two well separated peaks with instrument limited widths (resolutions *t*/Δ*t* ∼ 110) consistent with the existence of only two predominant dianion species in the gas phase (see ref. 46 and 47 for discussion of instrument performance and resolution).

Experimental collision cross-sections derived from the arrival times of isomers **1** and **2** in pure N_2_ buffer gas are 305 ± 10 and 299 ± 10 Å^2^, respectively, determined from the Mason-Schamp equation and instrument parameters (pressure, temperature, and arrival times corrected for time the ions spend outside of the drift region) – see ESI† for details.^69^,^70^ The experimental cross sections are consistent with ATD isomer **1** being associated with PO_4_,PO_4_ (calculated collision cross section 309 Å^2^) and ATD isomer **2** being associated with N-3,PO_4_ (calculated collision cross section 293 Å^2^). These assignments might be considered as tentative because collision cross sections calculated using MOBCAL rely on potential energy parameters that are not benchmarked for interactions between N_2_ and anions and assume static structures. Below in Section 3.2, we show that the assignments are consistent with the photo-responses of the two isomers.

The low energy N-3,PO_4_ and PO_4_,PO_4_ deprotomer structures described above are ‘open’ with minimal interaction between the adenosine and isoalloxazines units. For the N-3,PO_4_ deprotomer a proton is shared between adjacent oxygen atoms of the two PO_4_ units. The lowest energy π-stacked conformations of the N-3,PO_4_ and PO_4_,PO_4_ deprotomers lie 20 and 7 kJ mol^–1^ above the respective open conformations (see ESI†). For the π-stacked N-3,PO_4_ deprotomer the increase in energy may be due to Coulombic repulsion between the two negative charges. Calculated collision cross-sections for these π-stacked conformations are 297 and 291 Å^2^, respectively. Although our ATDs show evidence for only two gas-phase isomers with instrument-limited ATD peak widths, it is possible that the barriers between conformations are sufficiently low that open and stacked conformations interconvert rapidly during the ions' passage through the drift region leading to the appearance of a single peak for each deprotomer. For example, Gidden and Bowers found that deprotonated trinucleotides exhibited two ATD peaks at 80 K, assigned as open and folded conformations, while at higher temperatures (>200 K) only a single ‘time-averaged’ ATD peak was observed due to rapid interconversion between the conformations.^71^ If the same situation exists for FAD dianions, it may be difficult to compare experimental collision cross-sections, which represent conformationally-averaged structures, with calculated collision cross-sections that assume static structures.

Calculated energies of FAD dianions deprotonated on a phosphate and one of the hydroxyls along the ribityl chain lie higher in energy by 60–90 kJ mol^–1^ (see ESI†). Structures in which both phosphates are deprotonated and the N-3 hydrogen is moved to the N-1 or N-5 position lie higher in energy by >80 kJ mol^–1^. Structures deprotonated on the adenosine (sugar + adenine) unit also lie higher in energy (>80 kJ mol^–1^). None of these alternative deprotomers are expected to be important.

### 3.2 Action spectra of selected deprotomers

Both FAD deprotomers respond to visible light. Fig. 3(b) and (c) show ATDs recorded for the PO_4_,PO_4_ and N-3,PO_4_ deprotomers selected using ion gate IG2 (‘laser-off’, black trace) and ‘laser-on’–‘laser-off’ difference or photo-action ATDs (orange trace). The photo-action ATD for the PO_4_,PO_4_ deprotomer (Fig. 3(b)) shows a clear signature for photoisomerisation, with a depletion of the parent isomer signal and an increase in the signal at the expected position for the N-3,PO_4_ deprotomer (this assignment is confirmed in Section 3.3). The photo-isomer appears approximately half-way between the positions of the two deprotomers in panel (a), as the ions pass through the first IMS stage as the PO_4_,PO_4_ deprotomer and the second as the N-3,PO_4_ deprotomer. A minor, unassigned peak (isomer **3**) appears at even shorter arrival time. In contrast, the photo-action ATD for the N-3,PO_4_ deprotomer (panel (c)) shows only depletion with no discernible photoisomerisation. For both deprotomers, only parent FAD monoanions were observed when scanning the QMF, evidence that the net depletion is due to electron detachment rather than dissociation. The same situation pertained for RB and FMN monoanions for which no photofragment ions were observed, indicating that any depletion is due to electron detachment (see ESI† for ATDs).

**Fig. 4:**
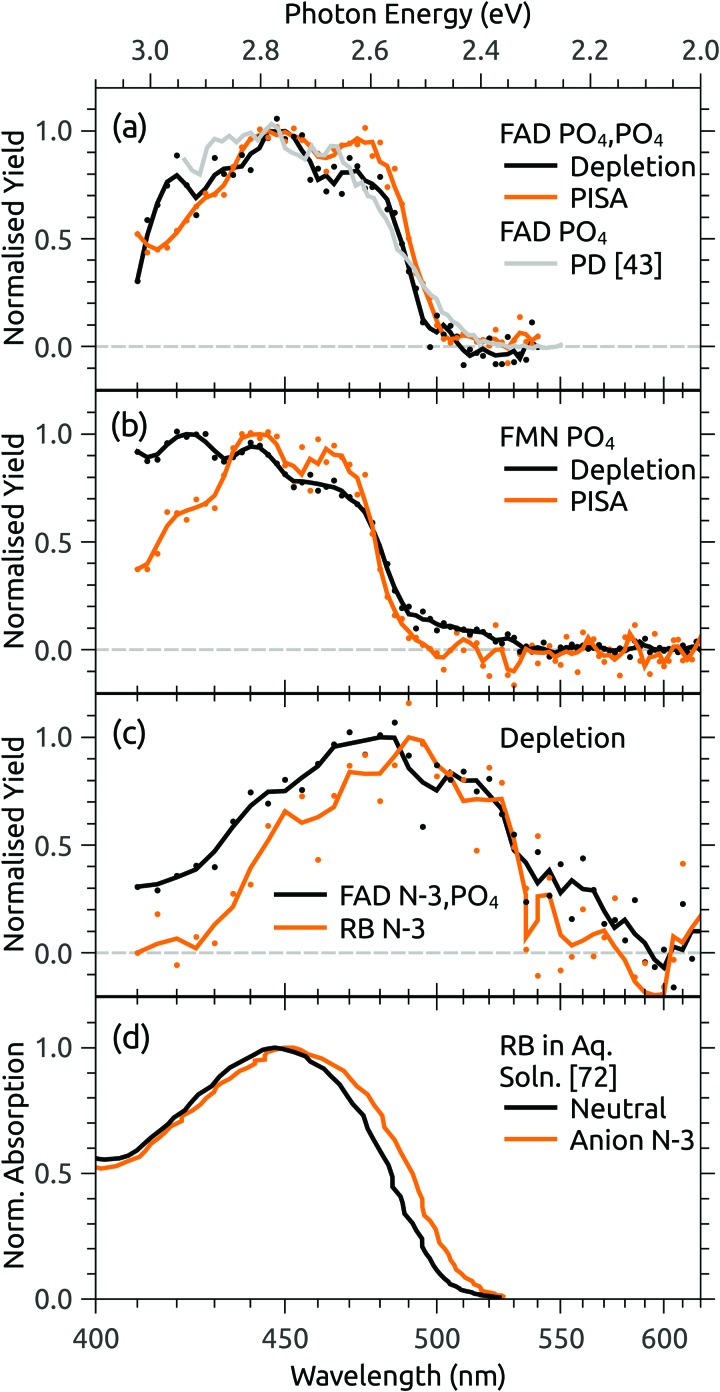
Action spectra for (a) FAD PO_4_,PO_4_ deprotomer and a photodissociation (PD) action spectrum for FAD monoanions from ref. 43, (b) deprotonated FMN monoanion, and (c) FAD N-3,PO_4_ deprotomer and deprotonated RB monoanion. Panel (d) shows normalised absorption cross-sections for RB in aqueous solution, adapted from ref. 72. Note that the PISA spectra in (a) and (b) have been multiplied by scaling factor 1.2 and 15, respectively.

Fig. 4 shows the ion depletion and photoisomerisation yields plotted as a function of laser wavelength, so-called ‘action spectra’. In panel (a), the depletion of the PO_4_,PO_4_ deprotomer and formation of the photo-isomer are associated with nearly identical action spectra, with the exception that the depletion signal exceeds the photoisomerisation signal by ∼30% with the difference due to electron detachment. Measurements of these spectra with lower laser power confirmed that the flat top was not due to saturation of the absorption band. For comparison, action spectra of FMN monoanions were also recorded (Fig. 4(b)). When electrosprayed from a sample of the phosphate sodium salt dissolved in dry acetonitrile (IF1 low), only one isomer associated with deprotonation on the phosphate group was observed. As for the FAD PO_4_,PO_4_ deprotomer, the photo-action ATD (see ESI†) and action spectra (Fig. 4(b)) show both depletion of the parent FMN signal, predominately due to electron detachment, but also minor photoisomerisation. The wavelength of maximum response is blue-shifted by ∼10 nm relative to FAD PO_4_,PO_4_ deprotomer, a shift also seen in the corresponding solution-phase absorption spectra.^21^ At this stage, we are unable to assign the FMN photo-isomer to a specific deprotomer.

Panel (c) of Fig. 4 shows the depletion (electron detachment) action spectrum for the FAD N-3,PO_4_ deprotomer. The wavelength of maximum response (∼485 nm) is red-shifted with respect to the PO_4_,PO_4_ deprotomer (panel (a), ∼450 nm), with a broad tail extending to 600 nm. For comparison, we also measured the depletion (electron detachment) action spectrum of deprotonated RB monoanion, which is included in panel (c) and closely resembles the spectrum of the FAD N-3,PO_4_ deprotomer. Deprotonation of RB on the isoalloxazine chromophore at the N-3 position (p*K*_a_ around 10) is expected based on studies in solution,^72^ and from our calculations of deprotomer energies (see ESI†). Panel (d) of Fig. 4 shows the normalised absorption cross-section for neutral and anion RB in aqueous solution, adapted from ref. 72. The anion spectrum is blue-shifted by ∼35 nm compared with the gas-phase spectrum in panel (c) due to the solvent interaction.

The assignment of two ATD peaks for FAD dianions to the PO_4_,PO_4_ and N-3,PO_4_ deprotomers is supported through the similarity of their respective action spectra with the monoanion spectra. A gas-phase photodissociation action spectrum of FAD monoanions (Fig. 4(a)),^43^ for which the location of deprotonation on one of the phosphates is not in doubt, is similar to the spectrum assigned to the FAD PO_4_,PO_4_ deprotomer, also supporting the assignment. Furthermore, the spectra assigned to the PO_4_,PO_4_ deprotomer resemble the absorption spectrum for neutral FAD in solution,^72^ consistent with deprotonation on both phosphates and minimal perturbation of the isoalloxazine chromophore.

Calculated transition wavelengths for the FAD PO_4_,PO_4_ and N-3,PO_4_ deprotomers at the df-CC2/6-31+G(d) level of theory are broadly consistent with the action spectra; for the PO_4_,PO_4_ deprotomer the S_1_ ← S_0_ transition is predicted to occur at 413 nm (oscillator strength 0.60) with a dark S_2_ ← S_0_ transition expected at 330 nm. The N-3,PO_4_ deprotomer is predicted to have red-shifted transitions at 469 nm (0.19) and 414 nm (0.24). The calculated adiabatic detachment energy for the N-3,PO_4_ deprotomer is ∼2.3 eV (∼540 nm), suggesting the depletion action spectra (electron detachment) for the FAD N-3,PO_4_ deprotomer and for deprotonated RB monoanions in Fig. 4(c) may ensue following absorption of a single photon (electron detachment can still occur for photons with energies below the adiabatic electron energy due to the additional internal energy of the ions at 300 K). A similar situation pertains for the FAD PO_4_,PO_4_ deprotomer for which the calculated adiabatic electron affinity is ∼2.8 eV (∼445 nm). The FMN monoanion with deprotonation on the phosphate group has a calculated adiabatic electron affinity of >4 eV, meaning that at least two photons are required for electron detachment for *λ* > 310 nm, although isomerisation may ensue following absorption of one photon. The difference between the deprotonated FMN monoanion depletion (multiphoton) and isomerisation (possibly single photon) spectra in Fig. 4(b) at shorter wavelengths may possibly be linked to changes in the probability for multiphoton absorption with wavelength.

Gas-phase dianions exhibit a repulsive Coulomb barrier (RCB) to electron detachment.^73^,^74^ From our minimum energy structures and the expression for RCB height from Wang *et al.*,^73^ we calculate the RCB to be 2.9 and 2.0 eV for FAD PO_4_,PO_4_ and N-3,PO_4_ deprotomers, respectively. Perhaps coincidentally, these values roughly correspond to the onsets for the depletion action spectra shown in Fig. 4(a) and (c). The depletion spectrum for the N-3,PO_4_ deprotomer has an onset at 600 nm (2.1 eV), whereas the PO_4_,PO_4_ deprotomer has a sharper onset at 500 nm (2.5 eV).

### 3.3 Discussion of intramolecular proton transfer

**Fig. 5:**
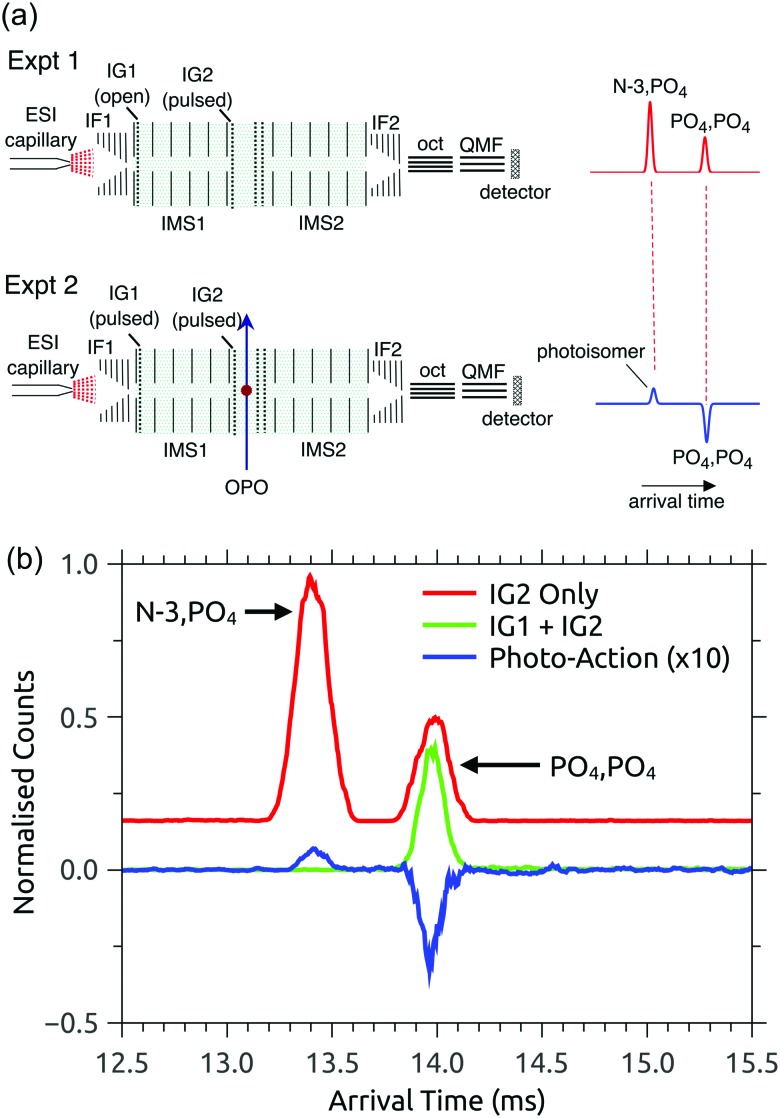
Assignment of the photo-isomer from the FAD PO_4_,PO_4_ deprotomer as the N-3,PO_4_ deprotomer: (a) experimental setups for measuring relative arrival times of the N-3,PO_4_ deprotomer (Expt 1, upper) and the photo-isomer from the PO_4_,PO_4_ deprotomer (Expt 2, lower), (b) arrival time distributions (N_2_ buffer gas seeded with ∼2% isopropyl alcohol) that suggest the photo-isomer formed following irradiation of the PO_4_,PO_4_ deprotomer is the N-3,PO_4_ deprotomer. ATDs are as follows: (i) red – using IG2 (IG1 disabled) to inject all electrosprayed ions into the IMS2 drift region, (ii) green – laser-off with IG1 operational and with IG2 gating the PO_4_,PO_4_ deprotomer, (iii) blue – photo-action from the PO_4_,PO_4_ deprotomer at 450 nm.

The PISA data shown in Fig. 3(b) and 4(a) demonstrate that the FAD PO_4_,PO_4_ deprotomer photo-converts to an isomer with a smaller collision cross-section. Although we cannot unequivocally identify this photo-isomer, we undertook the following procedure to demonstrate that the photo-isomer has the same arrival time (within ±0.02 ms) and relative collision cross-section (within ±0.5 Å^2^) as the N-3,PO_4_ deprotomer. These experiments were performed using N_2_ buffer gas seeded with ∼2% isopropyl alcohol dopant, which gave much better separation of the two deprotomers, such that their ATD peaks are baseline resolved. First, we sought to establish the relative arrival times for the N-3,PO_4_ deprotomer and the photo-isomer from the PO_4_,PO_4_ deprotomer. As shown in Fig. 5(a) upper, in the first experiment IG1 was opened completely to transmit all ions, while pulsing IG2. This allowed the deprotomers to separate over the second drift region (IMS2), yielding the red ATD shown in Fig. 5(b). In a second experiment (Fig. 5(a), lower), IG1 was pulsed injecting both deprotomers which separated as they traversed IMS1. IG2 was opened at an appropriate delay with respect to IG1, to select the PO_4_,PO_4_ deprotomer (green ATD in Fig. 5(b)), which was exposed to a pulse of visible light, generating the photo-isomer peak at 13.40 ms (blue photo-action ATD in Fig. 5(b)). Comparison of the blue and red traces shows that the photo-isomer peak has an arrival time within 0.02 ms of the N-3,PO_4_ deprotomer, confirming that they have the same relative collision cross-sections (within ±0.5 Å^2^) and suggesting that they are indeed the same species. Further evidence for this assignment could be obtained by recording an action spectrum of the photo-isomer formed from the PO_4_,PO_4_ deprotomer and comparing it with the action spectrum of the PO_4_,PO_4_ deprotomer. In principle, such an experiment would be achievable using a triple tandem IMS-IMS-IMS apparatus with provision for photoexcitation after the first and second stages.

Photoconversion of the FAD PO_4_,PO_4_ deprotomer to the N-3,PO_4_ deprotomer requires proton transfer between well separated sites. Two mechanisms initially come to mind: (i) an excited state proton transfer as proposed for FAD monoanions and protonated FMN cations,^39^,^43^ or (ii) recovery of the electronic ground state followed by statistical proton transfer(s), driving hot molecules to the more stable gas-phase deprotomer (N-3,PO_4_). Mechanism (i) presumably requires that the PO_4_,PO_4_ deprotomer can adopt a conformation in which the phosphates are located in the vicinity of the N-3 proton. However, our calculations were unable to locate a suitable minimum energy structure. Specifically, conformations in which an oxygen atom on the phosphate group closest to the adenine tail was constrained to have a 2.0 or 2.5 Å hydrogen bond with the N-3 proton were calculated to have energies 50–60 kJ mol^–1^ higher than the PO_4_,PO_4_ deprotomer; such conformations are unlikely to be accessed at room temperature. The reverse proton transfer, *i.e.* N-3,PO_4_ → PO_4_,PO_4_, *via* a similar conformation is probably even more unfavourable due to Coulombic repulsion between negative charges on the N-3 and PO_4_ groups. On the other hand, mechanism (ii) involves statistical proton transfer on the ground state manifold. There are two possible pathways. First, similar to excited state mechanism (i), there could be direct proton transfer between the two sites if the activated PO_4_,PO_4_ deprotomer samples a conformation in which the phosphates are close to the N-3 proton. In this instance, conformation sampling occurs on a vibrationally-hot ground state manifold such that conformations that are improbable at room temperature might be accessed. The second ground state pathway could involve a sequence of proton transfers along the ribityl chain (*e.g.* OH3 → PO_4_ then OH1 → OH3 followed by N-3 → OH1, see labeling convention in the ESI†), although this pathway seems unlikely as no evidence was found for any intermediate isomers in the photo-action ATDs. Molecular dynamics modeling may help ascertain if rearrangement on the ground state potential energy surface *via* the direct proton transfer pathway is competitive with collisional energy quenching in the ion mobility drift region, which is expected to occur over tens to hundreds of nanoseconds.^54^ Ultimately, further studies are needed to confirm the proton transfer mechanism.

### 3.4 Charge-transfer character for deprotonated RB

As noted earlier, the maximum in the absorption spectra of RB monoanions in solution (Fig. 4(d), see also ref. 72 and 75–78) is blue-shifted from the maximum for the gas-phase anions (Fig. 4(b)) by ∼35 nm, a shift attributable to a solvent effect. The S_1_ ← S_0_ transition of neutral flavins represents a textbook ππ* excitation^79^ that shows almost no solvatochromism.^78^,^80^,^81^ Deprotonation of the chromophore clearly alters the character of this transition, presumably by localising charge density in the vicinity of the deprotonation site. Electronic transitions showing significant solvatochromism or other micro-environmental sensitivity often have a high degree of charge transfer (CT) character.^56^ Energies of CT transitions may be strongly influenced by specific interactions with solvent molecules,^82^ counter-ions,^83^ or a protein micro-environment. On the other hand, ions with highly symmetric charge distributions have been shown to be nearly unaffected by such interactions.^27^,^84^ The observed red-shift in the absorption spectrum of the deprotonated flavin chromophore upon desolvation suggests that this transition has significant CT character.

To test the CT hypothesis for deprotonated isoalloxazine, we performed additional photodissociation measurements, allowing comparison of the action spectrum of deprotonated RB monoanion with that for complexes of deprotonated RB monoanion with the betaine (trimethylglycine, (CH_3_)_3_N^+^CH_2_CO_2_^–^) zwitterion. Betaine has a dipole moment exceeding 11.9 D^85^ and binds strongly to ions that have localised charge density in their ground electronic states. If electronic excitation moves charge density away from the binding site (*i.e.* if it is a CT transition), the charge-dipole interaction increases the energy cost and thus induces a blue-shift.^56^ Little or no spectral shift is observed for ions with highly delocalised charge distributions and no CT character.^56^

**Fig. 6:**
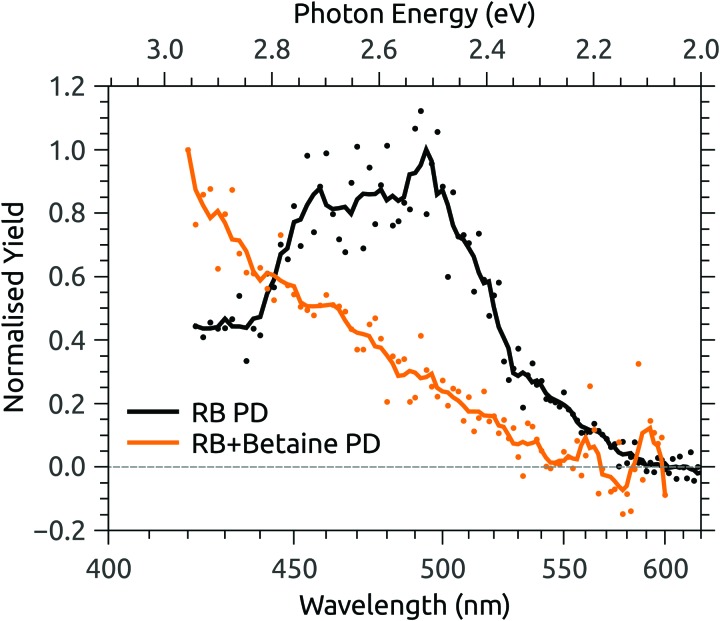
Photodissociation (PD) action spectra for deprotonated riboflavin (RB) monoanion and the complex of deprotonated RB monoanion and betaine.

Fig. 6 shows the photodissociation action spectrum for deprotonated RB monoanions (*m*/*z* 375) recorded by monitoring the photo-induced yield of deprotonated lumiflavin monoanion (*m*/*z* 255), the dominant photoproduct (see ESI†), as a function of laser wavelength. The band maximum and width are similar to the photodepletion spectrum recorded for the same species using the IMS instrument (Fig. 4(c)). Also shown in Fig. 6 is the action spectrum for the complex of deprotonated RB monoanion and betaine, recorded by monitoring photodissociation of the complex. The band maximum is beyond the tuning range of the available laser system, but the blue-shift induced by the addition of betaine is no less than 75 nm (0.45 eV), indicative of a high degree of CT character in the transition.^56^

**Fig. 7:**
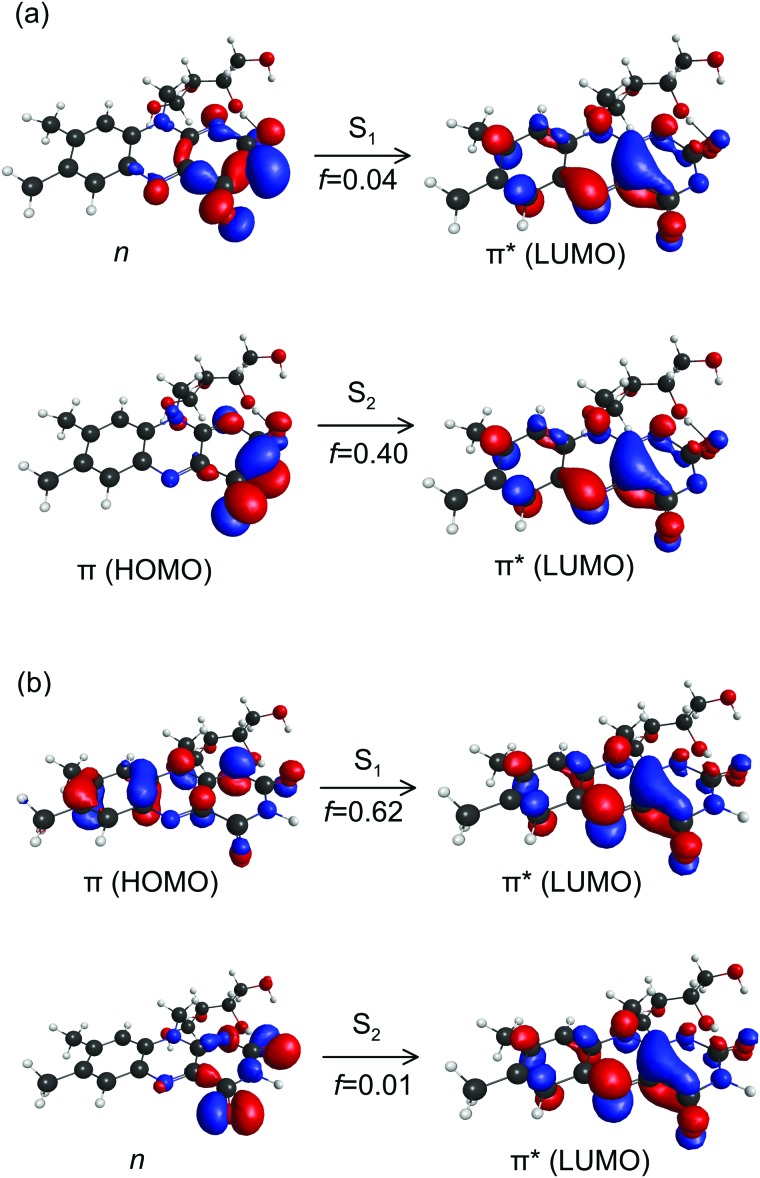
Canonical molecular orbitals associated with the S_1_ ← S_0_ and S_2_ ← S_0_ transitions for: (a) N-3 deprotonated RB monoanions, and (b) neutral RB molecules. For (a), both transitions have strong CT character. For (b), the dark S_2_ ← S_0_ transition has CT character. *f* are calculated oscillator strengths.

The CT character of the band is consistent with the predominant molecular orbitals associated with the S_1_ ← S_0_ and S_2_ ← S_0_ transitions for N-3 deprotonated RB monoanions (Fig. 7(a)). Specifically, our calculations show the S_1_ ← S_0_ transition has nπ* character with an oscillator strength of 0.04, whereas the S_2_ ← S_0_ transition has ππ* character with a larger oscillator strength of 0.40. Both transitions have strong CT character as they involve migration of electron density from the electronegative portion of the isoalloxazine group (*i.e.* localised around the carbonyl groups) to the opposite end of the chromophore. Similar CT transitions are expected for the S_1_ ← S_0_ and S_2_ ← S_0_ bands of the FAD N-3,PO_4_ deprotomer due to similar N-3 deprotonation. In contrast, the bright S_1_ ← S_0_ transition for RB monoanions deprotonated on the ribityl chain and the FAD PO_4_,PO_4_ deprotomer are expected to involve orbitals similar to those for neutral RB (Fig. 7(b)) which have minimal CT character.

## 4 Conclusions

The photochemistry of selected FAD deprotomer dianions has been probed by exciting the ions in a tandem IMS with tunable laser radiation. Two FAD deprotomers were observed, one in which both phosphate groups are deprotonated (PO_4_,PO_4_ deprotomer), and the other in which the isoalloxazine group and one of the phosphates are deprotonated (N-3,PO_4_ deprotomer). Photoexcitation of the PO_4_,PO_4_ deprotomer led to either electron detachment or an isomeric interconversion that appears to be proton transfer to form the N-3,PO_4_ deprotomer. Whereas photodepletion and photoisomerisation action spectra associated with the PO_4_,PO_4_ deprotomer closely resembled the absorption spectrum of neutral flavins in solution, the band of the N-3,PO_4_ deprotomer is red-shifted by ∼35 nm relative to the analogous band of the molecule in solution. This is interpreted as evidence that the lowest energy optical transition of flavin chromophore anions deprotonated on the isoalloxazine moiety possesses significant charge transfer character. This work illustrates the utility of tandem IMS action spectroscopy in unravelling the photochemistry of complex biochromphores. It also serves as a benchmark for quantum chemical calculations for flavins, and as a baseline for understanding the micro-environmental sensitivity of their optical transitions.

## Conflicts of interest

There are no conflicts to declare.

## Supplementary Material

Supplementary information
