# Supplementary material for: Ion mobility action spectroscopy of flavin dianions reveals deprotomer-dependent photochemistry
Source: Phys Chem Chem Phys. 2018 Jul 9;20(29):19672–81. doi: 10.1039/c8cp03244k (PMC6063075; doi:10.1039/c8cp03244k)
Supplement: Supplementary file 1 [file CP-020-C8CP03244K-s001.pdf]

Electronic Supplementary Material (ESI) for Physical Chemistry Chemical Physics.
This journal is © the Owner Societies 2018

Electronic Supplementary Information (ESI) for: Ion mobility action spectroscopy of flavin dianions reveals deprotomer-dependent photochemistry

James N. Bull[a], Eduardo Carrascosa[a], Linda Giacomozzi[b], Evan J. Bieske[a], and Mark H. Stockett[ab*]
[a] School of Chemistry, University of Melbourne, Melbourne, VIC 3010, Australia
[b] Department of Physics, Stockholm University, Stockholm, Sweden
* E-mail: mark.stockett@fysik.su.se

Calculated energies, transition wavelengths and collision cross-sections

Calculated energies, transition wavelengths and collision cross-sections for deprotomers of riboflavin (RB) monoanion and FAD dianion are given in Table S1 and Table S2, respectively. See Fig. S1 for the deprotomer labelling convention. Calculation of the $S_2 \leftarrow S_0$ vertical transition wavelength for the deprotonated lumiflavin anion, which is structurally equivalent to riboflavin with the $-C_5O_4H_{11}$ tail replaced with a $-CH_3$ group, was 456 nm (oscillator strength 0.20) at the df-CC2/6-31+G(d) level of theory and 463 nm at the df-CC2/6-311++G(d,p) level of theory, suggesting the transition wavelength is approximately converged with basis set size.

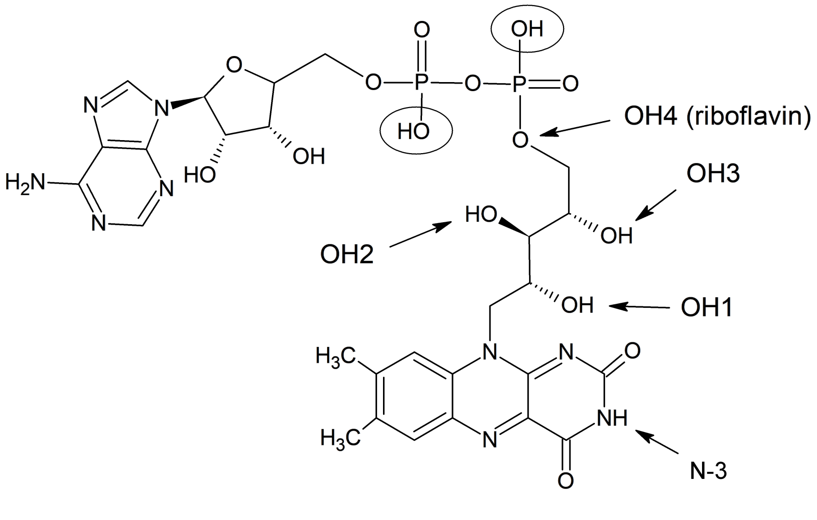

Figure S1 Labelling convention for deprotomers of FAD dianions and RB monoanions. The circles indicate deprotonation sites on the phosphate groups. For the N-3,PO_4 and OHx,PO_4 (x = 1-3) deprotomers, the remaining phosphate proton is shared between both PO_4 groups. Site OH4 is not relevant for FAD.

Deprotonation site	Relative energy (kJ/mol)	Transition wavelength (nm)	Collision cross-section (Å^2)
N-3	0	S_1 = 518 (0.04), S_2 = 437 (0.40)	193
OH1	19	S_1 = 410 (0.54), S_2 = 346 (0.003)	199
OH2	19	S_1 = 420 (0.57), S_2 = 339 (0.009)	197
OH3	4	S_1 = 411 (0.59), S_2 = 347 (0.004)	202
OH4	5	S_1 = 411 (0.57), S_2 = 350 (0.004)	203

Table S1 Calculated relative energies, vertical transition wavelengths (df-CC2/6-31+G(d) level of theory, oscillator strengths in parentheses) and collision cross-sections in N_2 buffer gas for deprotonated riboflavin (RB) monoanions. Note: energies are given relative to the N-3 deprotomer.

Deprotonation sites	Relative energy (kJ/mol)	Transition wavelength (nm)	Collision cross-section (Å^2) (in N_2 buffer gas)
N-3,PO_4 – isomer **2**	0	S_1 = 469 (0.19), S_2 = 414 (0.24)	293
PO_4,PO_4 – isomer **1**	2	S_1 = 413 (0.60), S_2 = 330 (0.004)	309
OH1,PO_4	63	–	294
OH2,PO_4	86	–	296
OH3,PO_4	89	S_1 = 415 (0.60), S_2 = 343 (0.03)	322
N-3,PO_4 π-stacked	20	S_1 = 487 (0.51), S_2 = 434 (0.13)	292
N-3,PO_4 π-tee	74	S_1 = 468 (0.18), S_2 = 408 (0.24)	296
PO_4,PO_4 π-stacked	9	S_1 = 410 (0.60), S_2 = 328 (0.03)	297
PO_4,PO_4 π-tee	14	–	296

Table S2 Calculated relative energies, vertical transition wavelengths (df-CC2/6-31+G(d) level of theory, oscillator strengths in parentheses) and collision cross-sections for the lowest energy FAD dianion deprotomer structures – see illustrations of selected structures in Figure S2. Details for π-stacked conformations for the N-3,PO_4 and PO_4,PO_4 deprotomers are given in the lower section of the table. Efforts to locate a PO_4,PO_4 deprotomer in which one PO_4 group was closer to the N-3 hydrogen (i.e. for direct proton transfer) ultimately optimised to a PO_4,PO_4 structure in which the ribityl chain is directed away from the flavin unit.

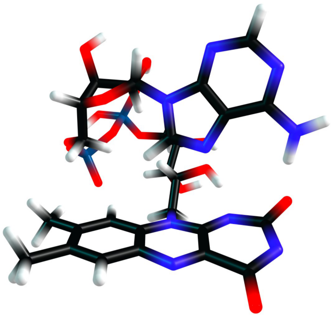

N-3, PO_4 - isomer **2**
E = 0 kJ/mol

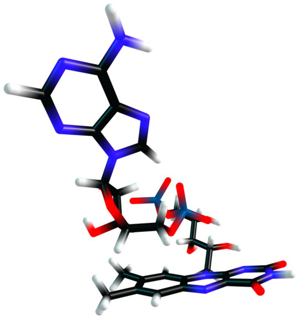

PO_4, PO_4 - isomer **1**
E = 2 kJ/mol

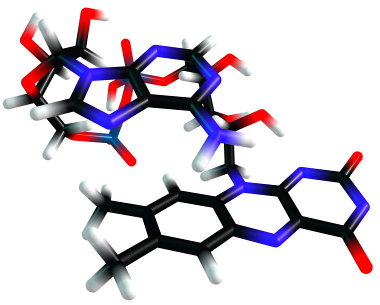

N-3, PO_4 π-stacked
E = 20 kJ/mol

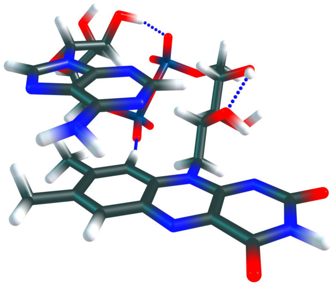

PO_4, PO_4 π-stacked
E = 9 kJ/mol

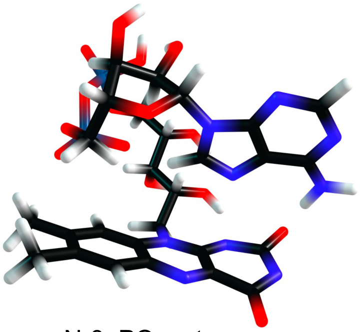

N-3, PO_4 π-tee
E = 74 kJ/mol

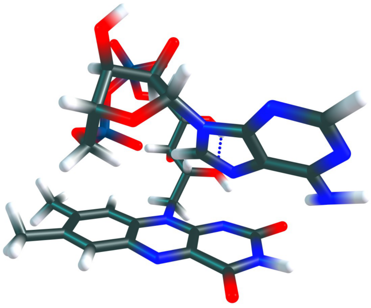

PO_4, PO_4 π-tee
E = 14 kJ/mol

Figure S2 Optimised structures for N-3,PO_4 (left column) and PO_4,PO_4 (right column) deprotonated FAD dianions. All energies are relative to the 'open' N-3,PO_4 deprotomer (top left).

Experimental collision cross-sections

Experimental collision cross-sections for the two FAD dianion ATD peaks in N_2 buffer gas (Fig. 3(a) in the paper) were determined using the Mason-Schamp equation and the relevant instrument parameters:

$$K = \frac{3ze}{16N}\sqrt{\frac{2\pi}{\mu k_B T}}\left(\frac{1}{\Omega}\right) = \frac{l^2}{t_d V}$$

Here, K is the ion's mobility, z is number of elementary charges carried by the ion (2 for FAD dianions), e is the electronic charge, N is the number density of the buffer gas (pressure measured using a calibrated Baratron gauge was 6.7±0.1 Torr), m is the reduced mass of the colliding ion-neutral pair, k_B is the Boltzmann constant, T is the absolute temperature, Ω is the collision cross-section, l is the length of the drift region (0.99 m including IF2), t_d is the transit time through the drift region and V is the potential drop across the drift region.

In our instrument the measured arrival time of an ion packet, t, is given by:

$$t = t_d + t_{oct} + t_{quad}$$

where t_d, t_{oct} and t_{quad} are the ion transit times through the drift region (6.7 ±0.1 Torr), octupole ion guide ($\approx 10^{-4}$ Torr), and quadrupole mass filter ($\approx 10^{-6}$ Torr), respectively. Values of t_{oct} and t_{quad} were calculated from instrument parameters (dimensions and kinetic energy of the ions in the octupole ion guide and quadrupole mass filter) and are small (≈0.3 ms) compared with t_d (≈12.2 ms).

For the two ATD peaks in Fig. 3(a) in the paper, values of t are 12.64 ms (isomer **1**) and 12.47 ms (isomer **2**). Corresponding values of t_d are 12.34 and 12.17 ms, respectively. Substituting these data into the Mason-Schamp equation yields Ω values of 305±10 and 299±10 Å^2, where the uncertainty is predominately associated with the buffer gas pressure. The uncertainty in relative collision cross-sections (assuming a ±0.02 ms uncertainty in t for each peak) is much less at ±0.5 Å^2.

It is difficult to compare directly the experimental collision cross-sections with the calculated values given in Table S2 due to the approximate nature of the MOBCAL approach and the lack of benchmarked parameters for interactions between N_2 and anions. Moreover, as discussed in the paper, there is probably rapid interconversion between FAD dianion conformations in the gas phase, meaning that the experimental cross-sections represent conformationally-averaged values whereas the calculations assume static structures. For example, the difference in energy between the 'open' and 'π-stacked' conformations for the PO_4,PO_4 deprotomer is only 7 kJ/mol, however the corresponding calculated collision cross-sections differ by 12 Å^2. The peak assigned to the PO_4,PO_4 deprotomer in our room-temperature ATDs is presumably associated with is a time-average of these and many other conformations.

FAD ATDs with isopropyl alcohol dopant

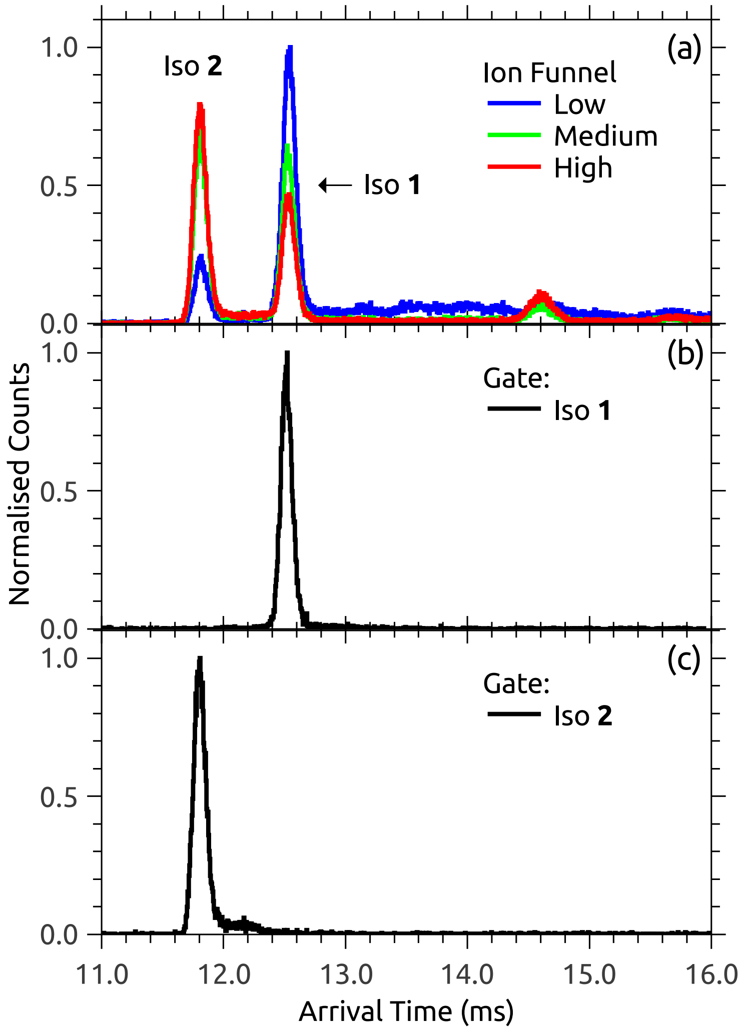

Figure S3 Arrival time distributions for FAD dianions using N_2 buffer gas seeded with ∼1% isopropyl alcohol: (a) FAD dianions under different ion funnel (IF1) conditions, and (b)/(c) isomers **1**/**2** gated with IG2. These ATDs show two well separated peaks with instrument limited widths (resolutions $t/\Delta t \sim 110$) consistent with the existence of two predominant dianion species in the gas phase. Note, the introduction of isopropyl alcohol dopant reduced the ion current making it difficult to collect action spectra.

Photo-action ATD for deprotonated FMN and RB monoanions

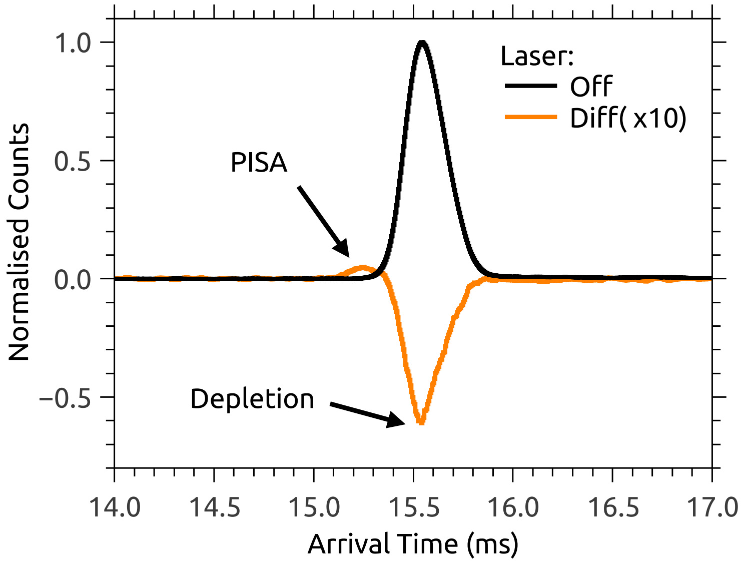

Figure S4 Arrival time distribution for deprotonated flavin mononucleotide (FMN) monoanions (black curve) and laser induced difference signal or 'photo-action' at 450 nm (orange curve). Both photodepletion and photoisomerisation are observed. The identity of the photo-isomer is not known, although we note that the calculated collision cross-sections for the PO_4 and N-3 deprotomers are 227 and 209 Å^2, respectively, suggesting that excitation causes conversion of the former to the latter. Note that an ATD without using IG2 was identical to the 'laser-off' ATD, which exhibits a single peak, consistent with the presence of only one isomer.

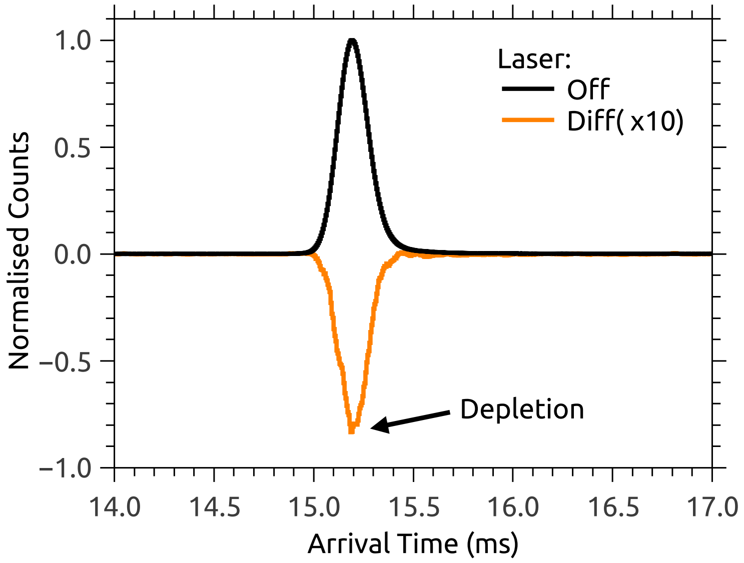

Figure S5 Arrival time distribution for deprotonated RB monoanions (black curve) and laser induced difference signal or 'photo-action' (orange curve) at 500 nm. Only depletion consistent with electron detachment was observed. Note that an ATD without using IG2 was identical to the 'laser-off' ATD, which exhibits a single peak with instrumentally limited width, consistent with the presence of only one isomer.

Photodissociation measurements

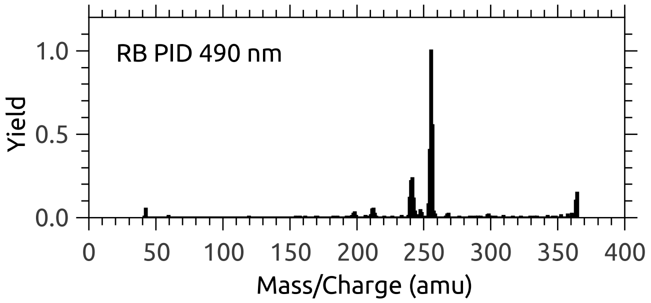

Figure S6 Photo-induced dissociation (PID) mass spectrum of deprotonated RB monoanions recorded using the SepI instrument at Aarhus University. The two main photofragments are deprotonated lumiflavin ($m/z = 255$) and deprotonated lumichrome ($m/z = 241$).

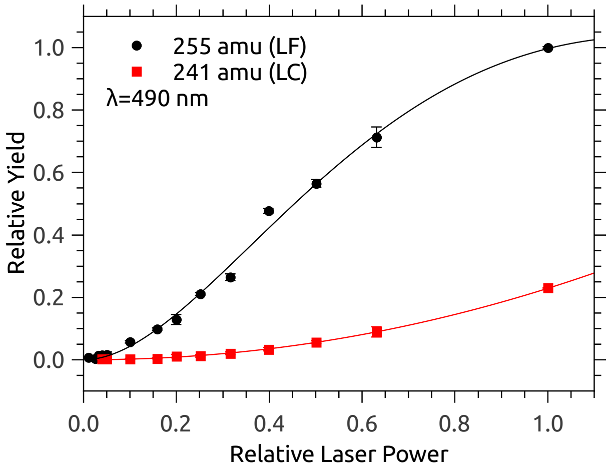

Figure S7 Laser power dependence of photo-fragment yields for deprotonated RB monoanions. Both yields are consistent with fits (solid lines) of the form $P^2 e^{-aP}$ (Poisson statistics). The P^2 factor indicates that the absorption of two photons is required to induce dissociation. LF = deprotonated lumiflavin, LC = deprotonated lumichrome.

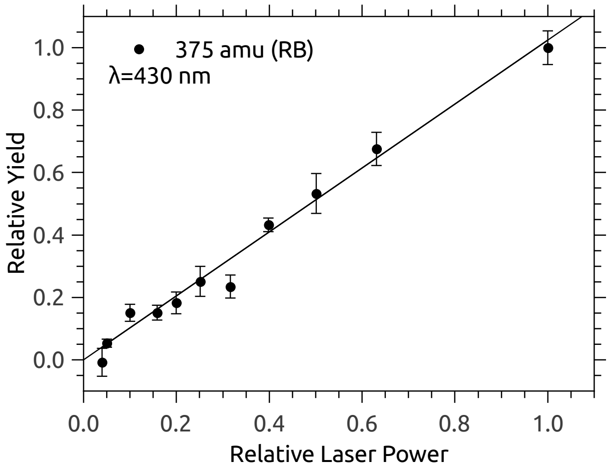

Figure S8 Laser power dependence of photodissociation yield for complexes of deprotonated RB monoanions and betaine. The yield is directly proportional to laser power, suggesting that a single photon induces dissociation. *m/z* 375 corresponds to the deprotonated RB monoanion.

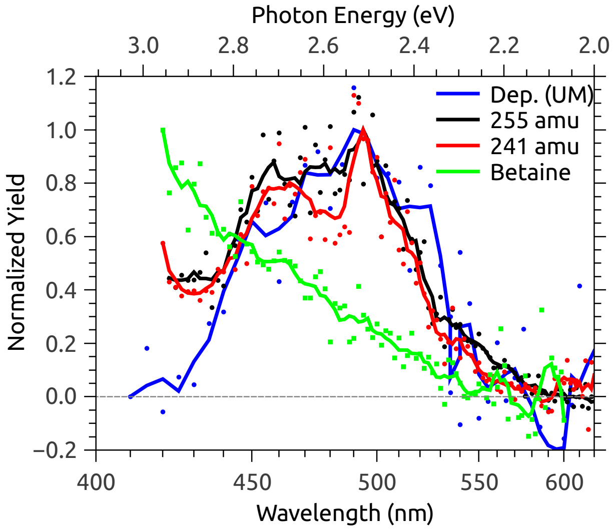

Figure S9 Comparison of action spectra for deprotonated RB monoanions recorded using the IMS instrument at the University of Melbourne (UM) and the SepI photodissociation instrument at Aarhus University. The action spectra of both main photo-fragments in the SepI experiments are identical, and both are very similar to the UM depletion spectrum. The addition of betaine induces a strong blue-shift.
